# Supplementary material for: A Novel Carotenoid with a Unique 2,6-Cyclo-ψ-End Group, Roretziaxanthin, from the Sea Squirt Halocynthia roretzi
Source: Mar Drugs. 2022 Nov 24;20(12):732. doi: 10.3390/md20120732 (PMC9784503; doi:10.3390/md20120732)
Supplement: Supplementary file 1 [file marinedrugs-20-00732-s001.zip › marinedrugs-2011089-supplementary.pdf]

Supplementary Materials

A Novel Carotenoid with a Unique 2,6-Cyclo-ψ-End Group, Roretziaxanthin, from the Sea Squirt *Halocynthia roretzi*

Takashi Maoka [1,*] and Chisato Tode [2]

[1] Research Institute for Production Development, Shimogamo-morimotocho, Sakyo-ku, Kyoto 606-0805, Japan
[2] Instrumental Analysis Center, Kobe Pharmaceutical University, Motoyamakita-machi, Higashinada-ku, Kobe 658-8558, Japan
* Correspondence: maoka@mbox.kyoto-inet.or.jp

Tables of Content

Figure S1. UV-VIS (Et_2O) spectrum of roretziaxanthin.
Figure S2. FAB MS spectrum of roretziaxanthin.
Figure S3. ^{1}H NMR spectrum ($CDCl_3$ 500 MHz) of roretziaxanthin.
Figure S4. ^{13}C NMR spectrum ($CDCl_3$ 125 MHz) of roretziaxanthin.
Figure S5. COSY spectrum ($CDCl_3$) of roretziaxanthin.
Figure S6. NOESY spectrum ($CDCl_3$) of roretziaxanthin.
Figure S7. HSQC spectrum ($CDCl_3$) of roretziaxanthin.
Figure S8. HMBC spectrum ($CDCl_3$) of roretziaxanthin.
Figure S9. CD spectrum (EPA) of roretziaxanthin.
Table S1 ^{1}H-NMR (500 MHz) and ^{13}C-NMR (125 MHz) of mytinoxanthin in $CDCl_3$.
Table S2 ^{1}H-NMR (500 MHz) and ^{13}C-NMR (125 MHz) of mytinoxanthinone in $CDCl_3$.
Table S3 ^{1}H-NMR (500 MHz) and ^{13}C-NMR (125 MHz) of halocynthiaxanthin in $CDCl_3$.

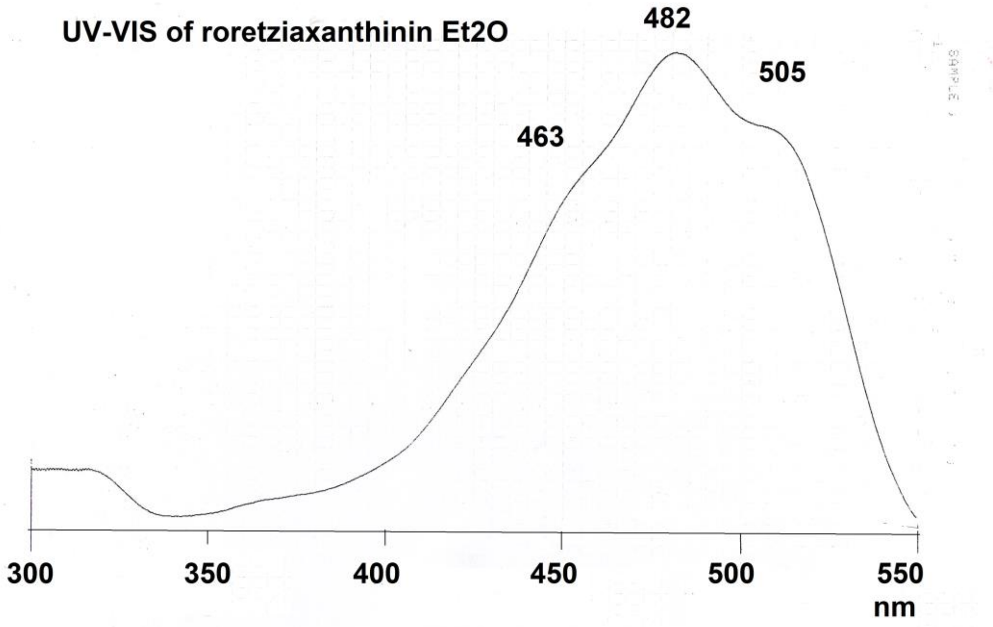

Figure S1. UV-VIS (Et_2O) spectrum of roretziaxanthin.

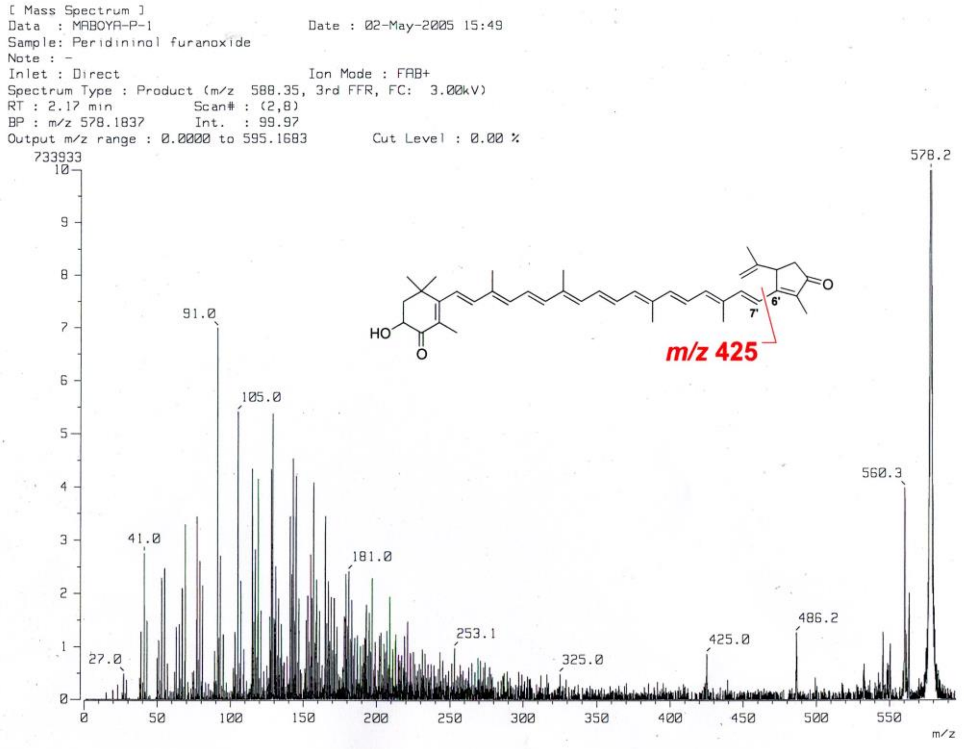

Figure S2. FAB MS spectrum of roretziaxanthin.

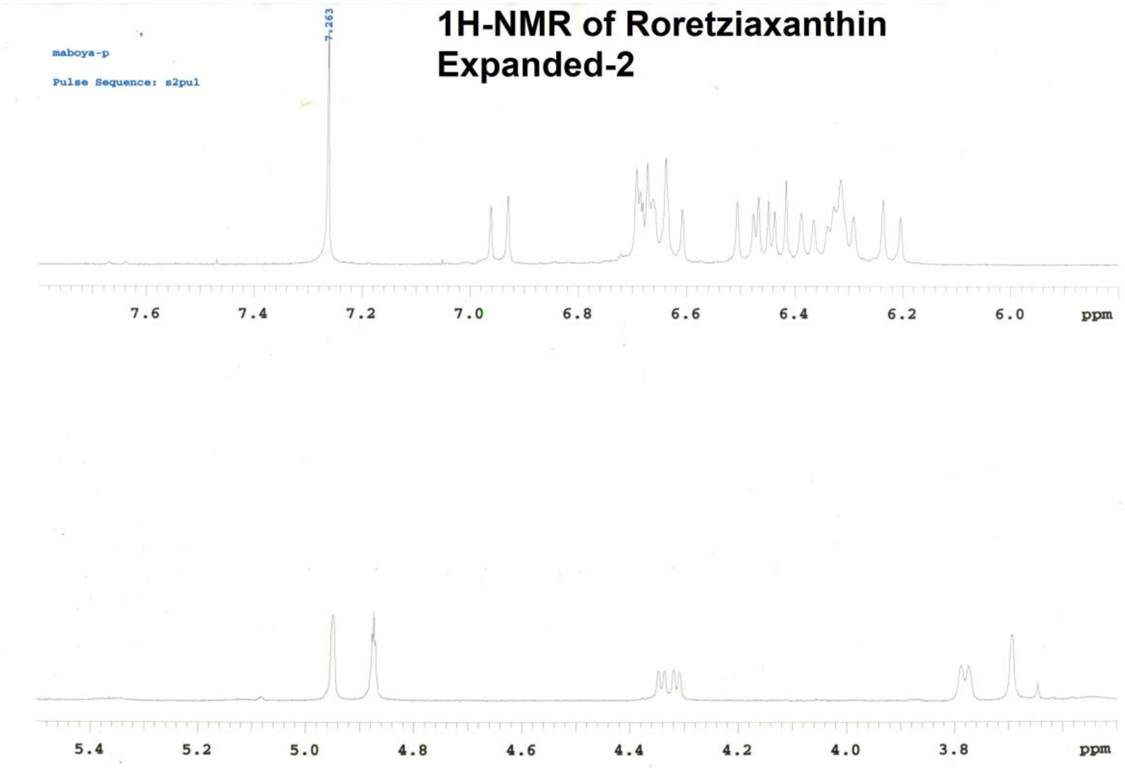

Figure S3. ^{1}H NMR spectrum ($CDCl_3$ 500 MHz) of roretziaxanthin.

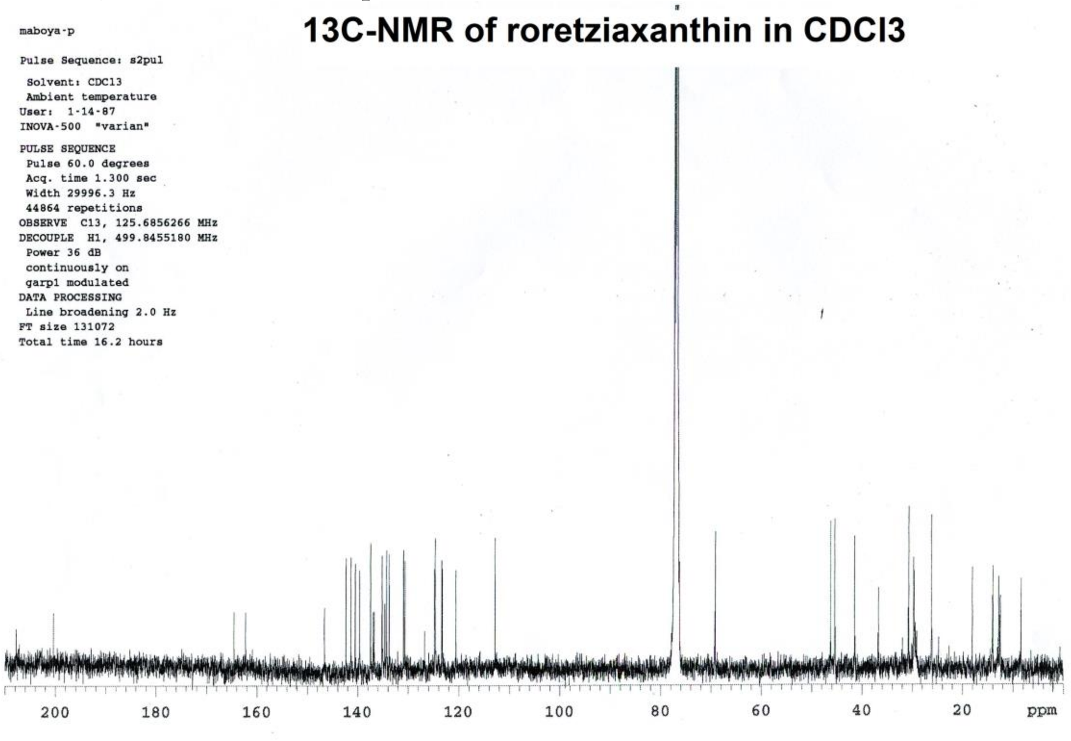

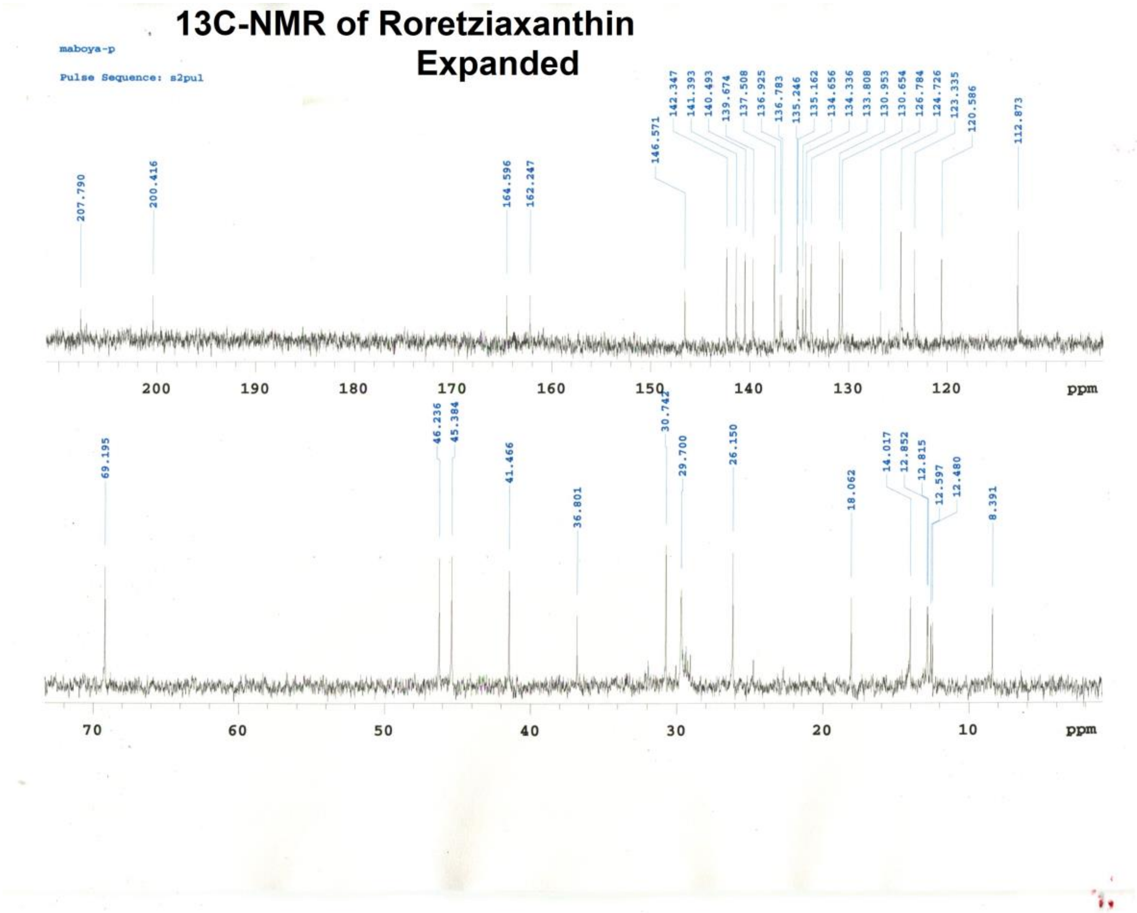

Figure S4. ^{13}C NMR spectrum ($CDCl_3$ 125 MHz) of roretziaxanthin.

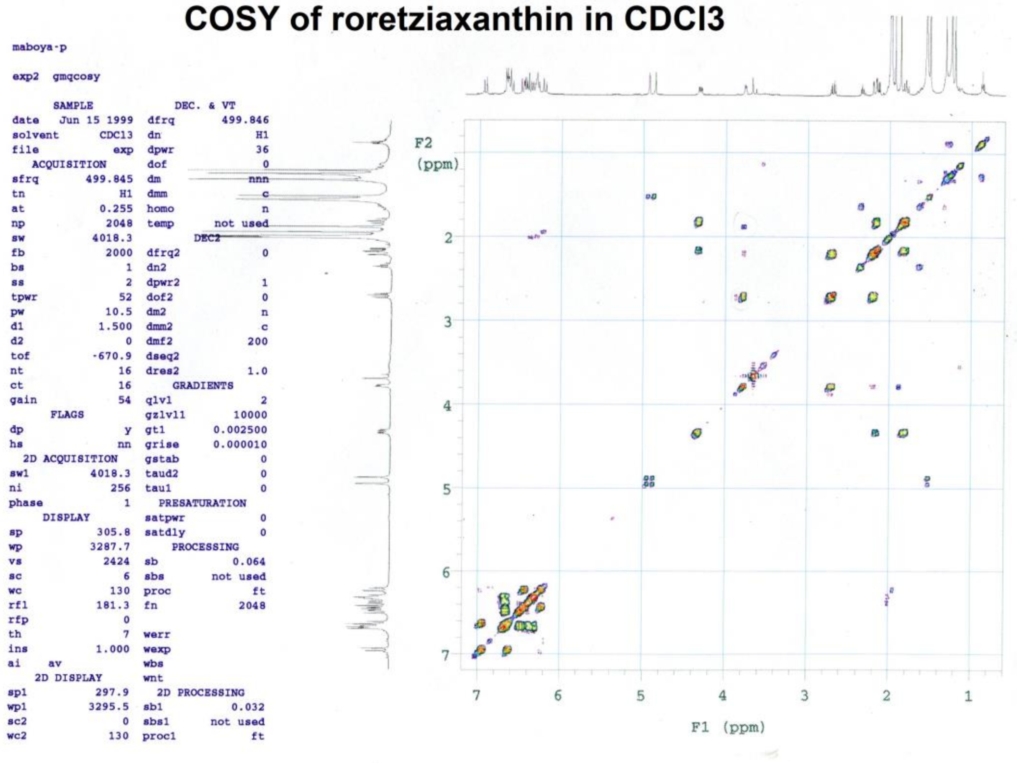

Figure S5. COSY spectrum ($CDCl_3$) of roretziaxanthin.

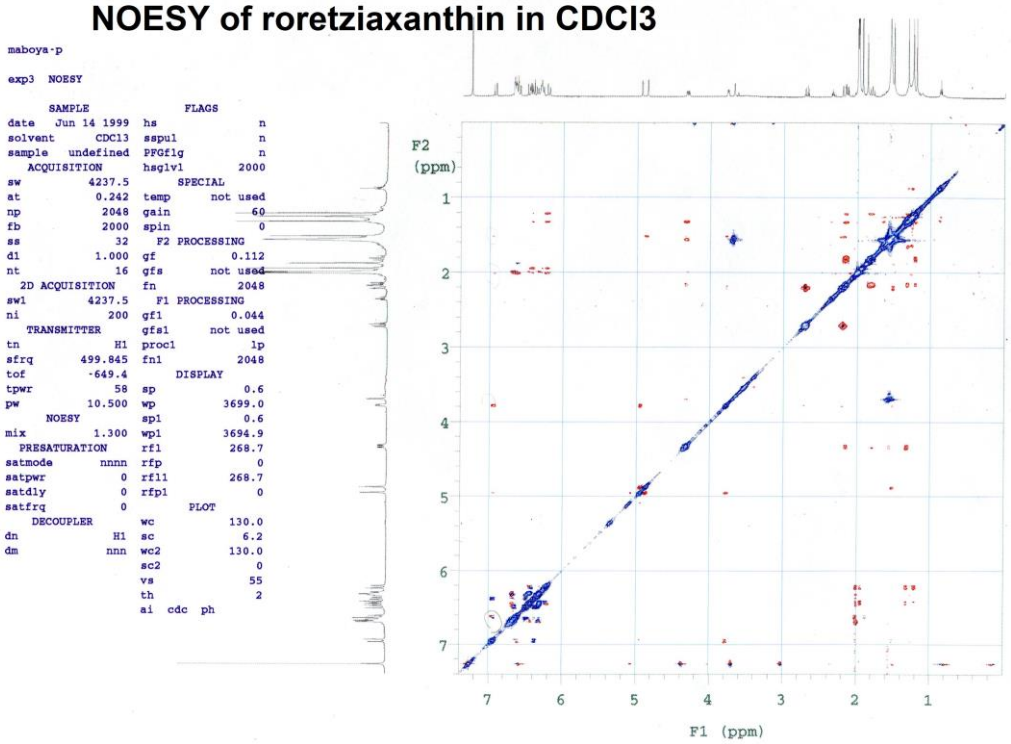

Figure S6. NOESY spectrum ($CDCl_3$) of roretziaxanthin.

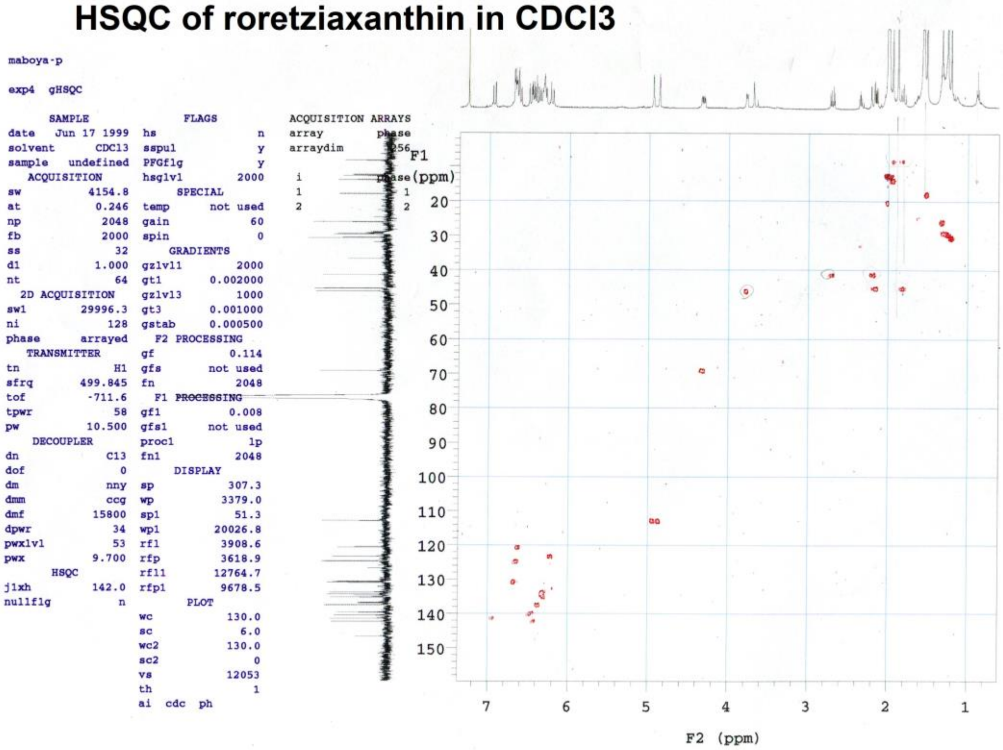

Figure S7. HSQC spectrum ($CDCl_3$) of roretziaxanthin.

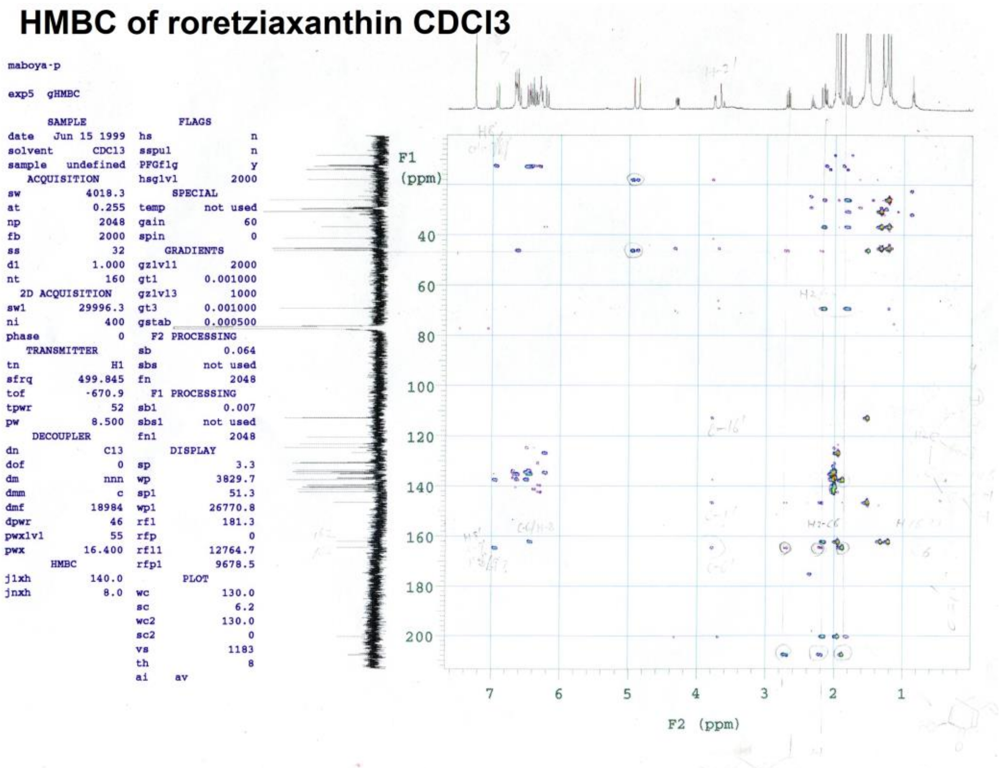

Figure S8. HMBC spectrum ($CDCl_3$) of roretziaxanthin.

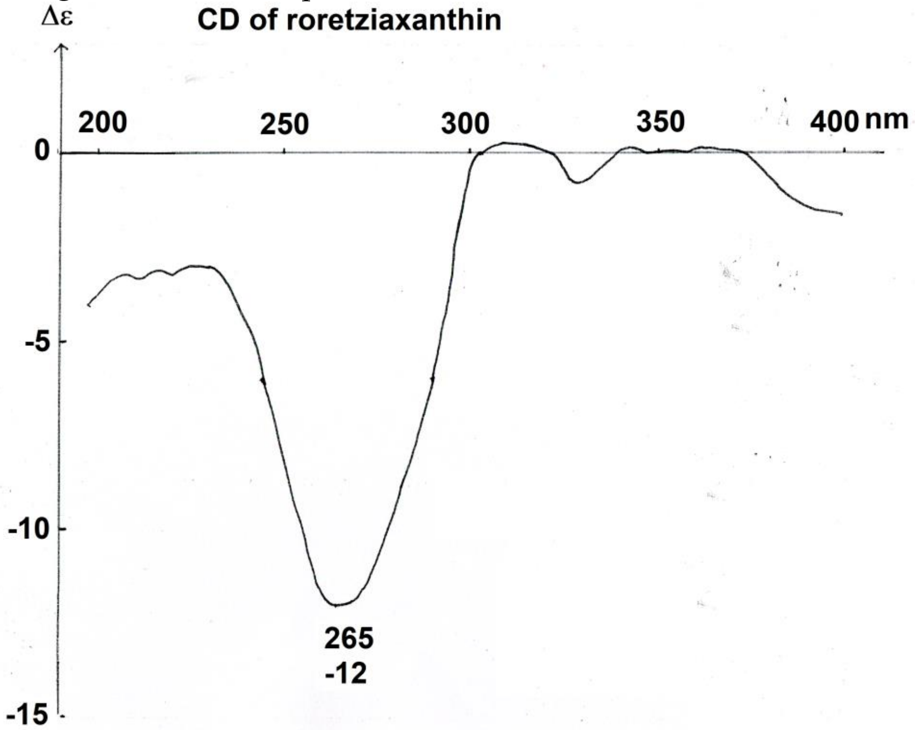

Figure S9. CD spectrum (EPA) of roretziaxanthin.

Table S1 ^{1}H-NMR (500 MHz) and ^{13}C-NMR (125 MHz) of mytiloxanthinone in $CDCl_3$.

**Positoin**	**^{13}C-NMR**	**^{1}H-NMR**		**Positoin**	**^{13}C-NMR**	**^{1}H-NMR**	
	δ	δ	mult. J (Hz)		δ	δ	mult. J (Hz)
1	36.60			1'	44.72		
2	46.70 ax	1.46	dd (12.0, 12.0)	2'	50.81 α	2.09	dd (13.5, 8.0)
	eq	1.84	ddd (12.0, 3.5, 2.0)		β	1.72	dd (13.5, 4.5)
3	64.87	3.99	m	3'	70.52	4.53	m
4	41.61 ax	2.07	dd (17.5, 9.5)	4'	45.22 α	2.88	dd (14.5, 8.5)
	eq	2.43	ddd (17.5,5.5, 2.0)		β	1.55	dd (14.5, 2.5)
5	137.46			5'	56.12		
6	124.91			6'	202.31		
7	89.32			7'	94.51	5.86	s
8	98.56			8'	182.00		
9	119.64			9'	136.41		
10	135.04	6.46	d (11.5)	10'	135.82	7.23	d (9.5)
11	124.22	5.54	dd (14.5, 11.5)	11'	123.60	6.62	d (15, 9.5)
12	137.89	6.36	d (14.5)	12'	144.00	6.65	d (15.0)
13	136.50			13'	137.80		
14	133.11	6.28	d (10.5)	14'	135.80	6.38	d (10.5)
15	130.00	6.70	dd (14.0, 10.5)	15'	132.60	6.65	dd (14.0, 10.5)
16	28.77	1.15	s	16'	25.91	0.85	s
17	30.50	1.20	s	17'	25.00	1.19	s
18	22.49	1.93	s	18'	22.21	1.35	s
19	18.09	2.01	s	19'	12.91	1.98	s
20	12.83	1.98	s	20'	12.83	1.99	s
				8'-OH		16.29	s

Table S2 ^{1}H-NMR (500 MHz) and ^{13}C-NMR (125 MHz) of mytiloxanthin in $CDCl_3$.

**Positoin**	**^{13}C-NMR**	**^{1}H-NMR**		**Positoin**	**^{13}C-NMR**	**^{1}H-NMR**	
	δ	δ	mult. J (Hz)		δ	δ	mult. J (Hz)
1	36.60			1'	41.49		
2	46.70 ax	1.46	dd (12.0, 12.0)	2'	52.38 α	2.40	d (18.0)
	eq	1.84	ddd (12.0, 3.5, 2.0)		β	2.23	d (18.0)
3	64.87	3.99	m	3'	216.53		
4	41.61 ax	2.07	dd (17.5, 9.5)	4'	48.30 α	2.99	d (18.0)
	eq	2.43	ddd (17.5,5.5, 2.0)		β	2.15	dd (18.0)
5	137.46			5'	53.12		
6	124.91			6'	200.34		
7	89.32			7'	94.10	5.86	s
8	98.56			8'	182.72		
9	119.64			9'	136.60		
10	135.04	6.46	d (11.5)	10'	135.82	7.23	d (9.5)
11	124.22	5.54	dd (14.5, 11.5)	11'	126.41	6.62	d (15, 9.5)
12	137.89	6.36	d (14.5)	12'	144.00	6.65	d (15.0)
13	136.50			13'	137.81		
14	133.11	6.28	d (10.5)	14'	135.96	6.38	d (10.5)
15	130.00	6.70	dd (14.0, 10.5)	15'	132.10	6.65	dd (14.0, 10.5)
16	28.77	1.15	s	16'	25.91	1.04	s
17	30.50	1.20	s	17'	20.50	1.23	s

18	22.49	1.93	s	18'	22.21	1.34	s
19	18.09	2.01	s	19'	12.91	1.98	s
20	12.83	1.98	s	20'	12.83	1.99	s
				8'-OH		16.26	s

Table S3 ^{1}H-NMR (500 MHz) and ^{13}C-NMR (125 MHz) of halocynthiaxanthin in $CDCl_3$.

**Positoin**	**^{13}C-NMR**	**^{1}H-NMR**		**Positoin**	**^{13}C-NMR**	**^{1}H-NMR**	
	δ	δ	mult. J (Hz)		δ	δ	mult. J (Hz)
1	36.60			1'	35.80		
2	46.70 ax	1.46	dd (12.0, 12.0)	2'	47.12 α	1.50	dd (12.0, 4.0)
	eq	1.84	ddd (12.0, 3.5, 2.0)		β	1.36	dd (12.0, 11.0)
3	64.87	3.99	m	3'	64.30	3.82	m
4	41.61 ax	2.07	dd (17.5, 9.5)	4'	41.72 α	2.33	dd (14.5, 8.5)
	eq	2.43	ddd (17.5,5.5, 2.0)		β	1.41	dd (11.0, 9.5)
5	137.46			5'	55.33		
6	124.91			6'	67.13		
7	89.32			7'	40.81	2.60	d (18.5)
						3.66	d (18.5)
8	98.56			8'	197.92		
9	119.64			9'	134.62		
10	135.04	6.46	d (11.5)	10'	139.11	7.15	d (11.0)
11	124.22	5.54	dd (14.5, 11.5)	11'	123.41	6.58	dd (15.5, 11.0)
12	137.89	6.36	d (14.5)	12'	145.22	6.67	d (15.5)
13	136.50			13'	135.61		
14	132.21	6.28	d (10.5)	14'	136.64	6.41	d (10.5)
15	132.51	6.70	dd (14.0, 10.5)	15'	129.42	6.65	dd (14.0, 10.5)
16	28.77	1.15	s	16'	25.11	1.04	s
17	30.50	1.20	s	17'	28.22	0.97	s
18	22.49	1.93	s	18'	21.21	1.22	s
19	18.09	2.01	s	19'	11.81	1.95	s
20	12.83	1.98	s	20'	12.83	1.99	s
